# A scoping review of national policies for healthy China from 2008 to 2024

**DOI:** 10.1186/s12889-026-26396-3

**Published:** 2026-04-14

**Authors:** Tingting Cai, Hongbin Yu, Yunliang Yao, Yunhui Xia, Zhou Zhou, Jiantong Shen, Jianlin Lou

**Affiliations:** 1 Medical School of Huzhou Normal University, Huzhou, 313000 China; 2 The Third Affiliated Hospital of Huzhou Normal University (Huzhou Third People’s Hospital), Huzhou, 313000 China; 3 Huzhou Key Laboratory for Precision Prevention and Control of Major Chronic Diseases, Huzhou Normal University, Huzhou, 313000 China

**Keywords:** China, Healthy China, Policy document, Policy review, Scoping review

## Abstract

This study reviews all relevant national policies related to the Healthy China Strategy from 2008 to 2024 and identifies potential policy gaps from a health systems perspective. A scoping review was conducted. Out of 7,591 policies, 306 were included. Essential data were extracted and mapped. Over 60 ministries were involved in policy-making, with multisectoral cooperation becoming a key trend. The National Health Commission and the National Administration of Traditional Chinese Medicine were identified as the primary leading ministries. To identify priorities and relationships, thematic content analysis was applied, which included the four systems, five reform pillars, and six domains. While several areas, such as leadership & governance, health workforce, and service delivery, received strong policy support, other components of the health system received limited coverage. Based on the findings, the following four policy recommendations are proposed: (1) facilitate multisectoral collaboration; (2) develop a unified policy system focused on Healthy China 2030; (3) strengthen health system building blocks, such as financing, health information, and medicine & technologies; (4) establish a comprehensive tracking, monitoring, and evaluation system.

## Introduction

Universal health coverage (UHC) is propelling the global health agenda and is a pivotal target of the United Nations’ Sustainable Development Goal 3 (SDG-3) [1, 2]. In 2023,world leaders made a new Political Declaration on “Universal Health Coverage (UHC): expanding our ambition for health and well-being in a post-COVID world”. This announcement was praised as a crucial impetus to achieve the UHC target of the Sustainable Development Goals (SDGs) by 2030 [3]. Low and middle-income countries (LMICs) were increasingly sharing their experiences from different political contexts to achieve UHC [4–7]. As a member of the United Nations, China is committed to achieving UHC [8–11]. As early as 2008, the Chinese government issued “Healthy China 2020”, which proposed 10 specific goals and 95 sub-goals [12]. Subsequently, in August 2016, President Xi Jinping placed health at the center of Chinese policy-making. He called for the full protection of every citizen’s health, stating “An all-around moderately prosperous society cannot be achieved without the people’s all-round health” [13, 14]. The Healthy China 2030 outline, the first medium- to long-term strategic plan for the health sector since the establishment of China in 1949, was formally released by the Central Party Committee and the State Council in October 2016 [13, 15]. This outline demonstrated China’s commitment to the UN 2030 Agenda for Sustainable Development. In July 2019, the Healthy China Action Promotion Committee issued the Healthy China Initiative (2019–2030), which included 15 special actions aimed at improving the health of vulnerable populations, such as women, children, and students, and preventing and controlling four major chronic diseases [16, 17]. The policy’s release was timely and momentous, given the increasing incidence of non-communicable diseases, poor eating habits, industrialization, urbanization, and a rapidly aging population. Soon after, the State Council issued “Implementation and Evaluation of the Healthy China Initiative”, providing a blueprint and action plan to facilitate the development of the Healthy China strategy [18]. In recent years, China has made huge progress, particularly in the areas of reproductive, maternal, neonatal and child health (RMNCH), non-communicable diseases, infectious diseases, and service capacity and access [19]. National statistics show that coverage of essential RMNCH services, which are either covered by social health insurance or financially subsidized, has reached high percentages comparable to those in high-income countries [19, 20]. For example, coverage for antenatal care and postnatal care reached 98.2% and 97%, respectively, while neonatal and infant mortality rates were around 2.8‰ and 4.5‰,respectively [21]. The mortality rate of tuberculosis has also been reduced to levels seen in high-income countries [19, 22]. However, the policy progress and potential gaps within the Healthy China Strategy remain unclear. This study primarily aims to characterize the national policies related to the Healthy China strategy and identify potential policy gaps from a health systems perspective. We have four specific objectives: first, to appraise the volume and variety of relevant policies from 2008 to 2024; second, to trace the evolution of policy-issuing ministry distribution; third, to identify the priorities and relationship among the key policies; fourth, to identify the strengths and potential gaps in existing policies. In addition to identifying areas for improvement in China’s policy-making process, this review will offer valuable lessons that could benefit other regional countries.

## Methodology

### Study protocol and registration

A scoping review was conducted using the guidance of the Preferred Reporting Items for Systematic reviews and Meta-analysis extension for Scoping Reviews (PRISMA-ScR) Checklist (Appendix 1) [23]. We reviewed national-level policies related to the Healthy China strategy from 2008 to 2024. The year 2008 was chosen as the starting point because it was the first calendar year after the Chinese government proposed the Healthy China strategy.

### Eligibility criteria

1. Inclusion criteria
  Issued by the government at the national level; and
  Published by the State Council and its 54 affiliated ministries; and
  Resolution, Decision, Note, Opinion, and Notice were the types of policy documents that qualified.

(2) Exclusion criteria
  Issued before 2008; or
  Not published in full; or
  Expired policies; or
  Policies restricted to a specific context, such as region-specific policies; or
  Interpretations or subsequent responses to prior policies; or
  Policies providing factual statements, such as technical standards or statistical data; or
  Not directly related to the six domains of the Healthy China strategy.

### Data sources

In March 2024, an online search was conducted through the publicly accessible PKULAW Database. (https://www.pkulaw.com/). The search included policies issued by the State Council and its 54 affiliated ministries (Appendix 2).

### Search strategy

A systematic search strategy was developed to gather policy documents [24]. Initially, we limited our policies issued at the central government level and restricted the presence of “healthy China” in the title. 41 core policies were obtained according to the previous search process. Based on the 41 policies, we synthesized related keywords (Appendix 3). Finally, we searched the policy titles from the PKULAW Database.

### Data extraction and screening

The extracted elements for each policy included policy title, policy-issuing ministry, policy-issuing date, and information related to the Healthy China and health system perspectives. One reviewer performed initial data extraction, and a second reviewer subsequently extracted data from a randomly selected sample (20%) of the documents to ensure accuracy and consistency.

### Analytical framework

We used a framework that integrated the Healthy China 2030 Plan and the WHO Health System Building Blocks [25, 26]. According to the Healthy China 2030 Plan, there are six domains in Healthy China strategy: (i) healthy life; (ii) health services; (iii) health security; (iv) healthy environment; (v) health industry; (vi) support & guarantee, which addresses “how to strategically optimize” the health system. The WHO Health System Building Blocks Framework contains six building blocks (service delivery, health workforce, health information, medicine & technologies, financing, leadership & governance), which indicates “what constitutes” a health system. Health systems could be successfully realigned to serve the Healthy China strategy with strong and all-encompassing policy support. The WHO health system framework offered a means for comprehending the components and structure of the health system.

### Synthesis of results

Data synthesis involved four steps. Firstly, the essential data were compiled and summarized. Secondly, the interconnection of policies was recognized. Thirdly, specific strategies or actions were inductively coded in these policies. Fourthly, the analytical framework was mapped to the content of the policy documents that were related to Healthy China.

## Results

**Fig. 1:**
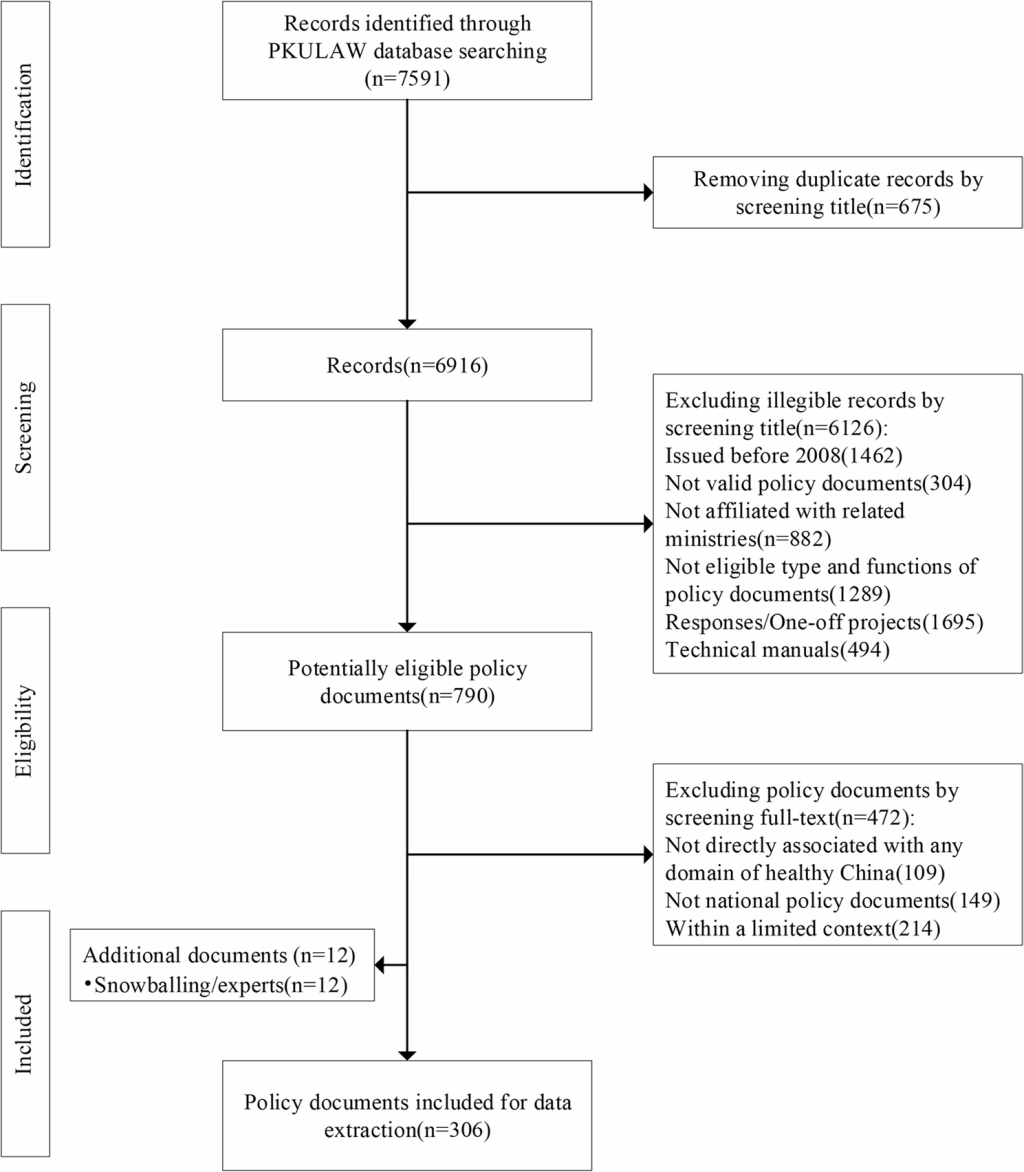
The flow chat of policy identification

Our search yielded 7591 policies from the PKULAW database (Fig. 1). After the removal of duplicate documents and full-text screening, a total of 306 eligible policies were collected.

### Volume and characteristics of eligible policies

**Fig. 2:**
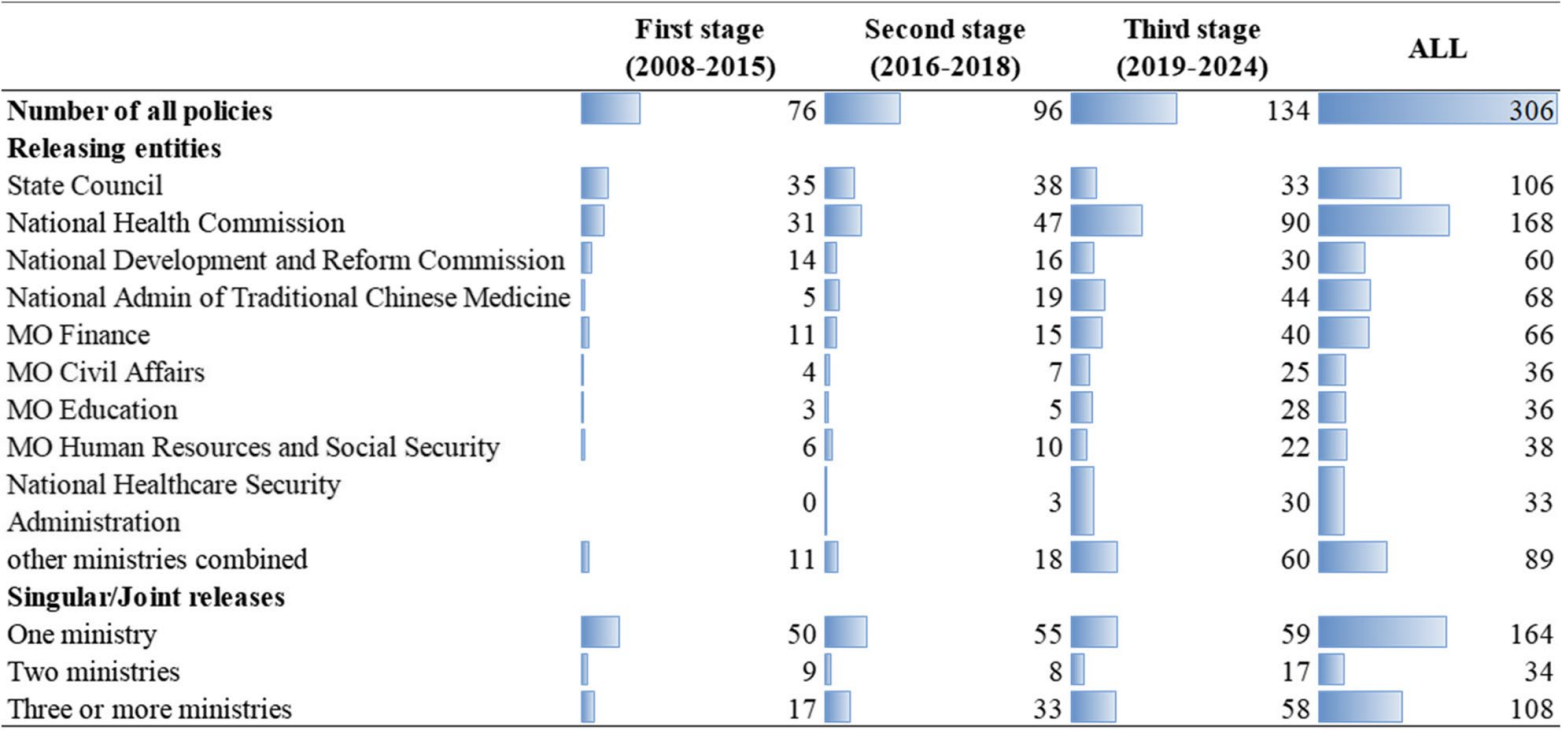
The number and variety of eligible policies by three time periods

Based on expert opinions and relevant literature on Healthy China policy research, this review was divided into the following three stages. We statistically analyzed the 306 policies to characterize the stage of policy releases, the ministry of releases, and the number of releases (Fig. 2). Over the past seventeen years, there has been a significant increase in the number of newly-issued policies, rising from 76 during 2008–2015 to 134 during 2019–2024. In the third stage, approximately half of the eligible policies (*n* = 134) were issued. The Chinese National Health Commission issued the largest number of policies, spanning nearly every stage (*n* = 168), followed by the State Council ranking (*n* = 106). More than half of the included policies (*n* = 164) were issued by a single ministry. Notably, the proportion of inter-ministerial jointly issued policy documents has shown a steady increase since 2016.

### Multisectoral collaboration in policy-making

**Fig. 3:**
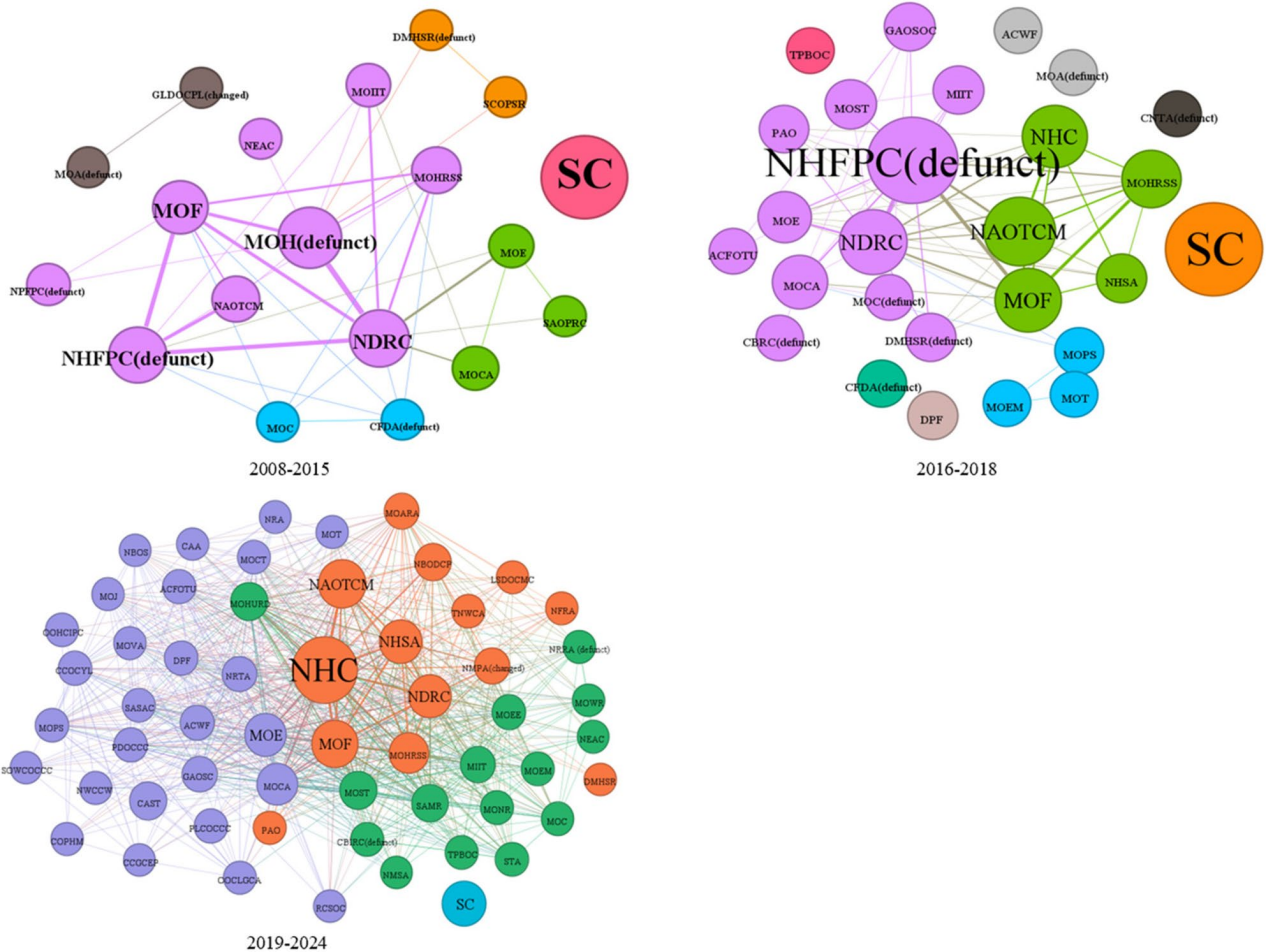
Evolution of interministerial collaboration in healthy china policy issuance, 2008–2024

Figure 3 illustrates a joint policy-issuing network across different stages based on collaborative relationships. Each node represents a ministry, and each edge indicates a joint policy-issuing relationship. From 2008 to 2015, the core entities of the joint policy-issuing network included the National Health and Family Planning Commission (defunct), the National Development and Reform Commission (defunct), and the Ministry of Health (defunct). These bodies played a central role in macro-level management and steering the development of China’s health system. From 2016 to 2018, multiple ministries began jointly issuing documents. While some ministries continued to issue policies independently, overall cooperation among ministries grew substantially. Health-related ministries gradually assumed a leadership role, reflecting the government’s efforts to comprehensively implement the Healthy China strategy. Between 2019 and 2024, the number of ministries involved in policy-making reached 57, with more than half of the policies jointly issued by at least two ministries. This demonstrates that joint policy-issuing became the norm and was increasingly systematic among government departments during this period.

### Leading policies and policy relationship

**Fig. 4:**
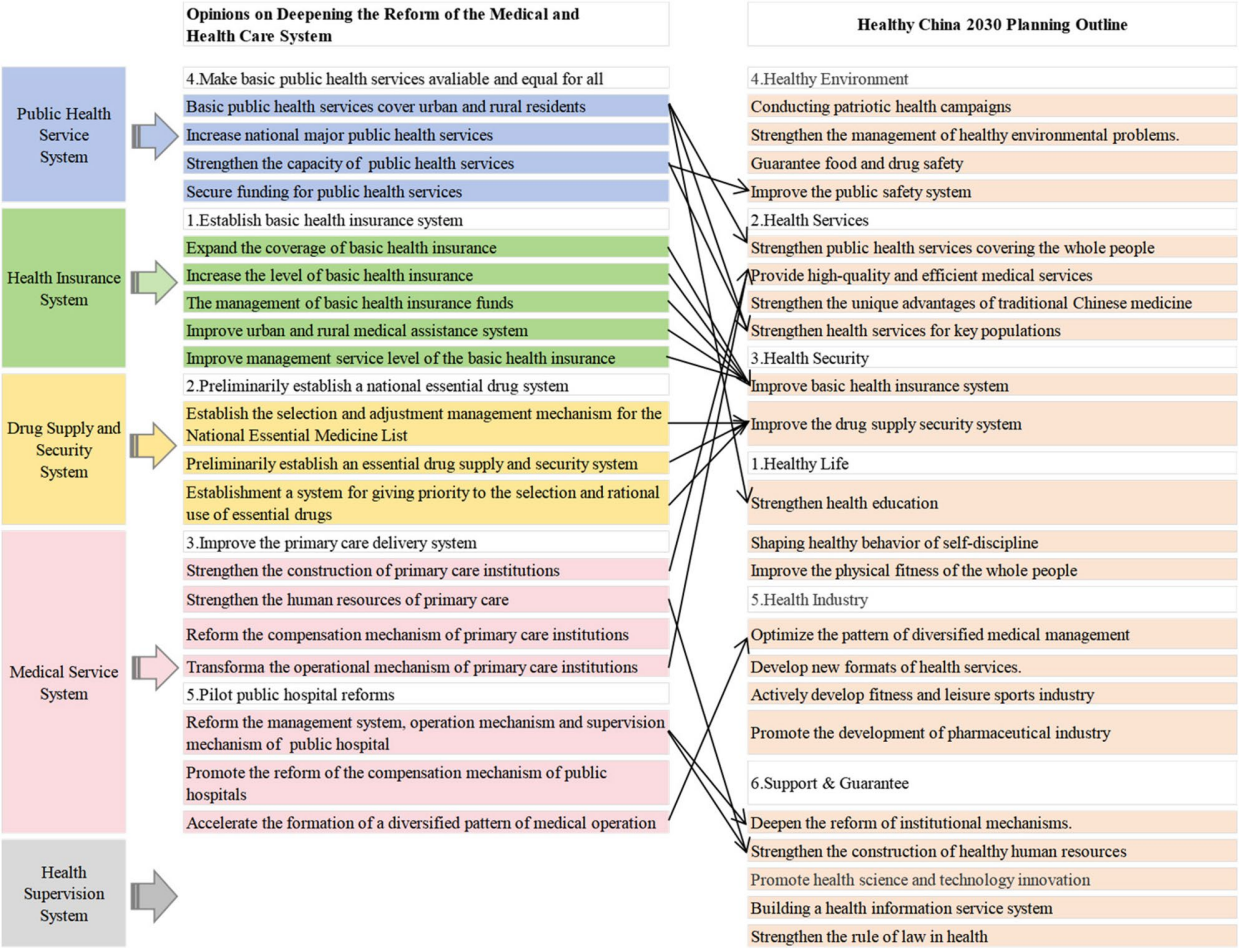
The major policy initiatives priorities and relationship among the leading policies

Figure 4 illustrates the priorities and relationships among the leading policies. The Chinese government launched a new round of health reform in 2009, aiming to achieve UHC by 2020. To achieve this goal, China focused on building four key systems: the public health service system, the medical service system, the health insurance system, and the drug supply and security system. Five reform priorities were further clarified, which laid a foundation for the 2020 goal. The Five reform priorities at this stage earned positive feedback and were validated by the WHO as moving in the correct direction. In 2016, the program for Healthy China 2030 was reviewed and approved by the Political Bureau of the CPC Central Committee, which set an action plan for the construction of a healthy China in the next 15 years. This phase is guided by the ‘Healthy China 2030’ strategy, which focuses on healthy life, health services, health security, healthy environment, and healthy industries, and the main proposals included upgrading hospital treatment and management, improvements in the supply of essential medicines, revitalization of traditional Chinese medicine, enhancement of health literacy, and stronger disease prevention. The policy primarily aimed to provide equitable, available, systematic, and continuous health services, focusing on the entire population and the whole life cycle.

### Key areas: mapping against the combined theoretical framework

**Fig. 5:**
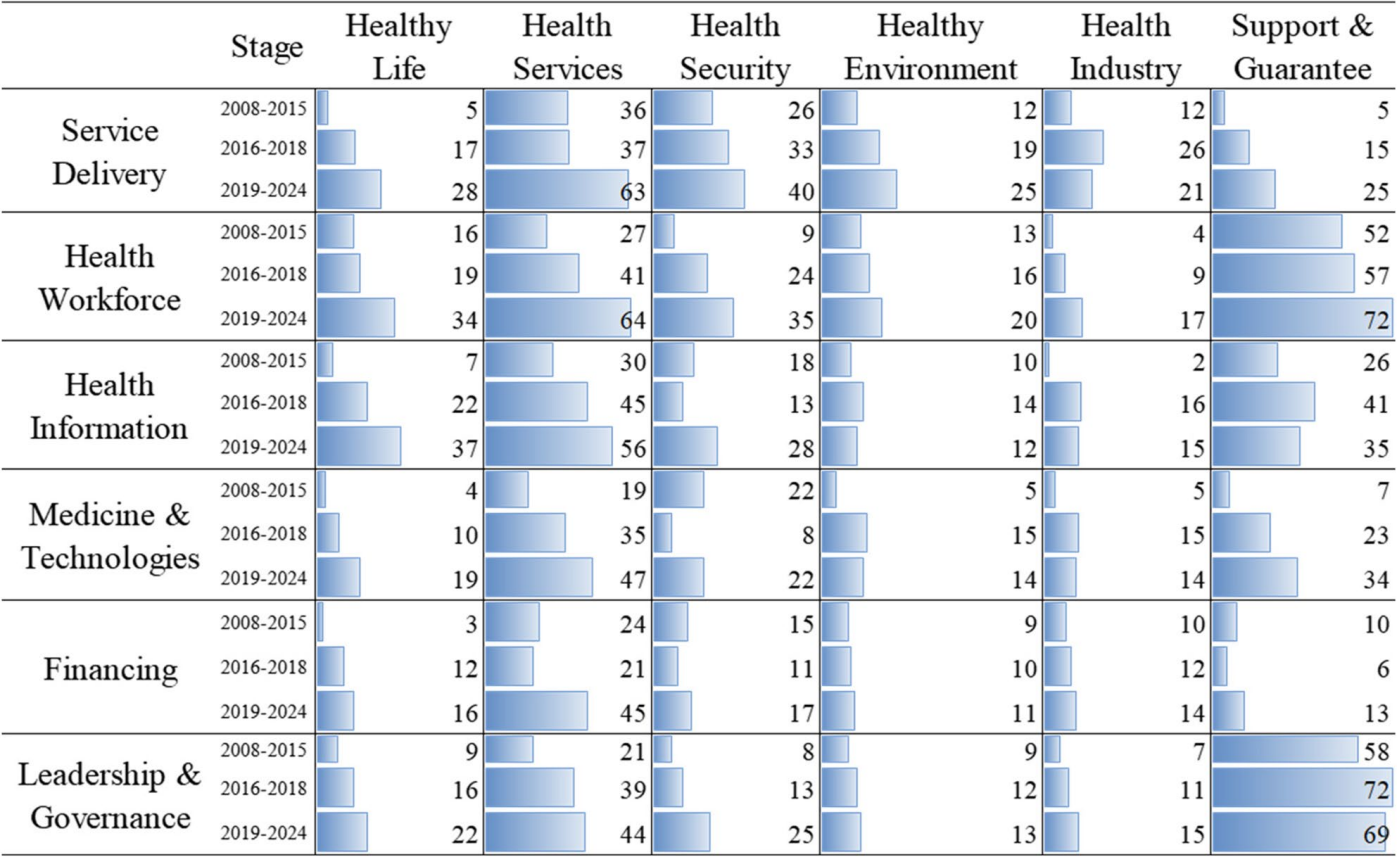
The combined theoretical framework is mapped against the number of policy documents

The number of policy documents has been increasing, covering the Healthy China domains (column) and health system building blocks(row) (Fig. 5). Based on the Healthy China domains, support & guarantee received the most policy attention (225 policy documents), followed by health services (153 policy documents), whereas ‘health industry’ was the least. Among these policies, the State Council and National Health Commission concerned all domains, while other ministries primarily focused on domains related to their respective administrative responsibilities. Based on the WHO Health System Building Blocks, policies referring to leadership & governance largely aimed to engage social forces in Healthy China strategy through simplifying administration, delegating powers, increasing regulation, and policy advocacy. At the health workforce level, key themes included expanding employment options, safeguarding employees’ legal rights and interests, providing in-service training and ongoing professional development, and enhancing incentive reform. At the financing level, policy documents focused on optimizing the efficiency of financial subsidy utilization, expanding potential financial sources, and regulating patients’ behavior through medical insurance payment. Establishing a nationwide compatible digital health information system was consistently highlighted as a priority in the health information system. At the service delivery level, a hierarchical healthcare mechanism was established, with clear roles and responsibilities assigned from primary care facilities to tertiary hospitals. Improved access to essential medicines & technologies promoted the development of essential medicine system, particularly for special population disease treatment and marginalized regions, while also driving innovation in medical industry advancement. All integrated components were addressed in existing policies within the combined analytical framework, but the extent of coverage differed. For example, health workforce and service delivery were largely covered by policies in each domain, especially in health services, whereas each one was sparsely addressed in the health industry.

## Discussion

This scoping review comprehensively analyzed national policies related to Healthy China from 2008 to 2024, demonstrating a huge political commitment to implementing the Healthy China strategy. Guided by the six domains and six building blocks, the review identified strengths and potential gaps in relevant policies, as well as uneven policy efforts across policy domains.

Over the past years, there has been an increase in the number of national policy documents that pertain to Healthy China, with nearly half of policies within the past five years. It is worth mentioning that the COVID-19 pandemic led to a slower pace of policy making, with an average of 32 policies per year from 2016 to 2018, compared to only 22 from 2019 to 2024. The prevention and control of the COVID-19 epidemic become the primary focus of healthcare policy during this period [27].

Approximately half of the policies were jointly issued by two ministries, with over 60 ministries involved in policy making. Recent policy reviews have identified a positive trend toward increased multi-sectoral collaboration [26, 28, 29], which supported the goal of multi-sectoral cooperation advocated by the WHO. However, the National Health Commission predominated in both singular and joint releases, followed by the National Administration of Traditional Chinese Medicine and the National Development and Reform Commission. These findings indicated that high-level coordination remained limited to a few selected ministries. With the development of Healthy China policies, success may depend on robust collaboration across relevant sectors under a strong coordinating authority, rather than the current system, to ensure effective implementation and impact [30].

Two leading policies and relationships were identified in this paper. First, the “Opinions on Deepening the Health Care System Reform” issued by the Central Committee of the Communist Party of China (CPC) marked a new round of health reform. This reform can be described as focusing on “four systems and five reform priorities”, providing a fertile ground to explore policy innovations in health reform [8, 9]. The two parts borrow and learn from the other in policy making, which leads to a mutually beneficial process. Second, regarding the leading policy addressing specific domains of Healthy China, we identified a progressive policy retention and adjustment process. For example, the basic health insurance and the essential drug system were integrated into the health security. However, considering the cross-cutting nature of the Healthy China strategy, making “health in all policies”workable and achievable is a highly complex mission. Thus, it is necessary to develop a unified policy system focused on Healthy China 2030 to minimize policy fragmentation and eliminate implementation barriers at various levels. Similar special task forces have already been established in other complex policy fields, such as education and energy [28]. 

In the Healthy China Plan, support & guarantee and health services were widely addressed. This facilitated national leadership in restructuring the healthcare system model, emphasizing task-shifting from tertiary hospital - centric frameworks to a hierarchical medical system [31, 32]. This was apparent in the essential medicine policy, the public health service package, and the basic health insurance schemes, all of which were designed to enhance the efficiency, accessibility, and affordability of services [30]. This was further proven by the increasing amount of primary care facilities and providers, as well as the steady rise in government-paid salaries [33, 34]. In addition, China’s unique poverty alleviation program has achieved a shift in goals from disease treatment to disease prevention and health protection. However, in contrast to the other domains, the healthy environment and health industry had the fewest supportive policies. Although the healthy environment has been headed by political leaders at every level of government, and involved in by various sectors and local communities, ensuring its sustainable remains a quite challenging issue. As a separate domain, the health industry had shown great importance from the Chinese Central Government since 2016. The health industry mainly focused on traditional industries, the intelligent retirement industry, structural supply-side reform, and the health service industry chain. Given the unique perspective of the health industry, there will be an opportunity to integrate the health industry into the public leisure life, to enrich and facilitate health needs, especially for older people.

From the health system perspective, a notable strength of the policy was its strong emphasis on leadership & governance. It played a critical role in steering the overall layout of a healthy China. In this policy review, we found that the essence of governance was to improve legislation work, transform government functions from direct providers to a supervisory role, encourage civil society and the private sector to participate in the development of private investment, and strengthen policy propaganda and interpretation [35]. Similar to leadership and governance, the health workforce was also a major focus. National policies inspired various financial support and technical support to deal with the current primary health care workforce shortage [26, 36]. Recent studies have indicated an urgent need to increase the number and quality of the health workforce to meet rapidly growing health demands [37, 38]. 

Unlike leadership & governance and health workforce, the remaining four blocks were poorly addressed in the existing policies. Although information development is recognized as a critical priority, the digitalization of the health information system and the “Internet+” action still face many obstacles due to inadequate system interoperability and limitations in data transfer [8]. China’s electronic health records market comprises over 300 vendors, each with their own proprietary technology structures and data standards [39]. Similarly, poor access to essential medicines, financing, and low-quality health services were identified as critical challenges in some studies [19, 26, 28]. For example, the availability of essential medicines was 4.29–43.75% in China, and much is to be done to achieve the goal of 80% availability suggested by the WHO [40, 41]. In future policy making, each ministry needs to overcome these longstanding issues.

This is the first scoping review focusing on national policies related to the Healthy China strategy. The study offers a thorough understanding of the national policies concerning healthy China by the Chinese Government in recent years. Some limitations in our study should be mentioned:1) We were limited to using open-source policies publicly available on the PKULAW Database. Although efforts to minimize significant omissions in policy documents through expert consultations, additional study is also required to include national and local policymakers in identifying potentially pertinent policies that remain undisclosed.;2) This study focused on national-level policies while neglecting policy differences and innovations among regions.;3) The extracted data in this review was not inadequate to assess the implementation and impact of these policies.

## Conclusion

In conclusion, our study provides an overall description of policies relevant to the Healthy China strategy in the past years moving towards UHC. However, compared with WHO recommendations, specific areas with scope for further policy strengthening in China were identified. First, facilitating multi-sectoral collaboration is necessary in both policy-making and execution processes. Second, a unified policy system focused on the Healthy China 2030 plan should be developed. Third, more proactive policy actions in health system building blocks, such as upgrading financial investment and funding management, establishing nationwide compatible digital health information systems, and promoting redistribution of resources across levels of care, including medicines, infrastructure, and technologies. Finally, the government should acknowledge the need for a robust performance evaluation system to support the implementation of the Healthy China strategy. The evaluation system should prioritize the quality of care and health outcomes to ensure their significant consideration. The experience of UHC in China can provide valuable lessons for other countries, particularly low- and middle-income countries (LMICs), that are planning their own paths towards UHC.

## Data Availability

No datasets were generated or analysed during the current study.
